# Editorial

**Published:** 1901-11-15

**Authors:** 


### EDITORIAL.

We have devoted considerable space the last three numbers of the Register to matters of general professional interest, because the past few months have been full of important events. The important business matters transacted by the National Association of Dental Faculties; the efforts made to put down the infamous traffic in dental degrees; and the effort made through the International Dental Federation to establish, if not a comity of relationship between the educational interests of all nations, at least an understanding as to what each country is endeavoring to do in the way of preparing men for the practice of dentistry. All these things seem to indicate a strong sentiment in favor of a higher standard of professional attainment in all countries, and the efforts being made to put this sentiment into practical form seem to be gathering new force each day. There seems to be a determination to make the new century one in which our science and art shall make even greater progress than during the last century, in which it was born and developed into a respected and even an honored profession. We have felt that it was our duty to encourage this spirit of progress in every way, believing that it will not only bring honor to us as a profession, but that each individual ought, for the sake of his profession and his own personal development, to take more than a passing interest in that which concerns the whole profession.

The St. Louis State Dental Society has appointed a committee which is to take the preliminary steps looking toward the formation of an International Dental Congress, which shall hold its meeting at St. Louis, during its World's Fair in 1903. The plan and scope of its work, as so far outlined, is similar to the work accomplished by the Chicago and Paris World's Fairs of 1893 and 1900. We have no doubt that an interesting and valuable meeting may be had by following this general plan. But would it not be wiser should some single purpose be made the objective point? As this great fair is planned to celebrate an historical event, why not make its Dental Congress an historical celebration. Let us have a grand celebration commemorating the works of our dead as well as living heroes. By a willing and concerted effort it would be possible in the time now at our disposal to gather such a complete history of our literature and professional development as may be impracticable at a future date. We all remember that something of this kind was attempted at the Columbian Dental Congress, but the work of this committee was so overslaughed by the more technical affairs, that the data which it did get together was never presented or even printed. But if the entire force of a great international congress could be directed at recording our history, we should have established once for all our history up to date. This should be a sufficiently worthy object for even a world's dental congress.

---

The thirty-sixth annual meeting of the Ohio State Dental Society will be held at the Great Southern Hotel in Columbus, Ohio, on the 3rd, 4th and 5th of December, 1901. Let everyone make arrangements to attend the meeting and bring along something which he has found interesting or helpful during the past year. The program committee will doubtless supply a full program of papers, etc. But clinics and personal experiences are the things which can be talked over in the recess hours, and they are invaluable helps in making acquaintances and forming friendship.

---

The Institute of Dental Pedagogics which meets at Pittsburg, December 31st, is one of the most interesting meetings of the year. All persons, whether teachers or not, if interested in teaching methods will be well paid for attending this meeting.

